# Supplementary material for: Nondestructive in-line sub-picomolar detection of magnetic nanoparticles in flowing complex fluids
Source: Sci Rep. 2018 Feb 22;8:3491. doi: 10.1038/s41598-018-21802-2 (PMC5823888; doi:10.1038/s41598-018-21802-2)
Supplement: Supplementary file 1 — Supplementary material [file 41598_2018_21802_MOESM1_ESM.pdf]

Supplementary materials to "Nondestructive in-line sub-picomolar detection of magnetic nanoparticles in flowing complex fluids"

Lykourgos Bougas[1,+,*], Lukas D. Langenegger[2,+], Carlos A. Mora[2], Martin Zeltner[2], Wendelin J. Stark[2], Arne Wickenbrock[1], John W. Blanchard[3], and Dmitry Budker[1,3,4,5]

[1]Johannes Gutenberg-Universität Mainz, 55128 Mainz, Germany
[2]Functional Materials Laboratory, Department of Chemistry and Applied Biosciences, ETH Zurich, CH-8093 Zurich, Switzerland
[3]Helmholtz-Institut Mainz, 55128 Mainz, Germany
[4]Department of Physics, University of California, Berkeley, CA 94720-7300, USA
[5]Nuclear Science Division, Lawrence Berkeley National Laboratory, Berkeley, CA 94720, USA
[*]lybougas@uni-mainz.de
[+]these authors contributed equally to this work

Magnetometer Calibration

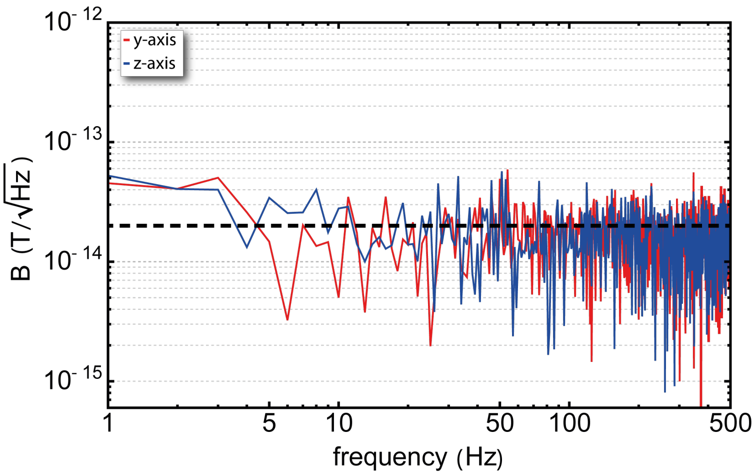

Figure S1: Noise measurements of the QuSpin magnetometer, for both magnetically sensitive magnetometer-axes (y & z). The shown calibrated magnetic field noise spectrum is measured inside the Twinleaf four-layer mu-metal co-axial cylindrical shield (Twinleaf; MS-2), that also includes compensation coils to remove ambient DC fields. The sensitivity of the magnetometer, averaged from 1 Hz to 200 Hz, is 17 fT/$\sqrt{\text{Hz}}$ for both y- and z-axis respectively. The emphasized gridline (black, dashed) corresponds to a 20 fT/$\sqrt{\text{Hz}}$ noise level.

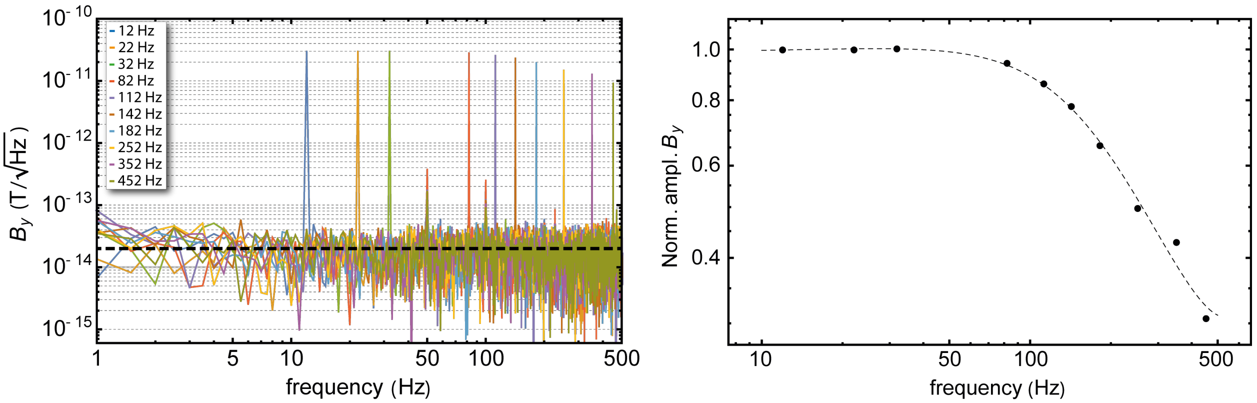

Figure S2: Calibration of the magnetometer as a function of the frequency of applied magnetic fields. (a) Uniform magnetic fields (30 pT in amplitude) at different frequencies are applied in the y direction to allow for the calibration of the magnetic field noise spectrum, and for the calibration of the measured voltages from the analog outputs of the magnetometer control module into magnetic fields. Note the observed power-line-related magnetic-field-noise frequency components at 50 Hz and 100 Hz. In (b), the frequency response of the magnetometer is clearly observed. The magnetometer acts as a low-pass filter for high-frequency magnetic field signals, with a cutoff frequency at ∼145 Hz.

C/Fe3C & C/Co Magnetic-Nanoparticle Properties

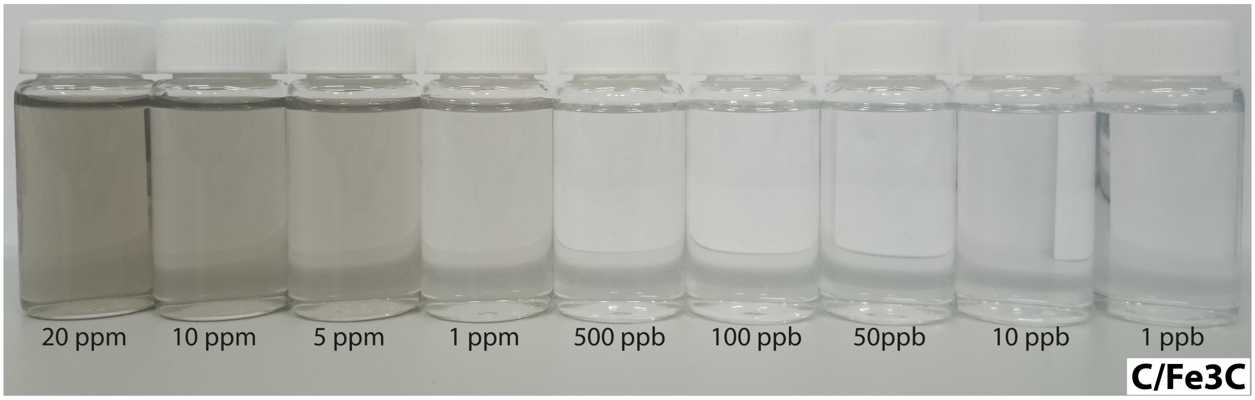

Figure S3: Photographs of the 20 ml water solutions of C/Fe3C nanoparticles used in our measurements.

Using inductively coupled plasma optical emission spectrometry (ICP-OES), we independently verify the concentration of different prepared solutions used in the measurements. The results are shown in Table T1 and Table T2.

Table T1: ICP-OES results for C/Co solutions

**C/Co**	
Sample Concentration (ppm)	ICP Concentration (ppm)
20	27.20
1	0.94
0.1	0.09

Table T2: ICP-OES results for C/Fe3C solutions

**C/Fe3C**	
Sample Concentration (ppm)	ICP Concentration (ppm)
10	7.99
1	0.86
0.1	0.12

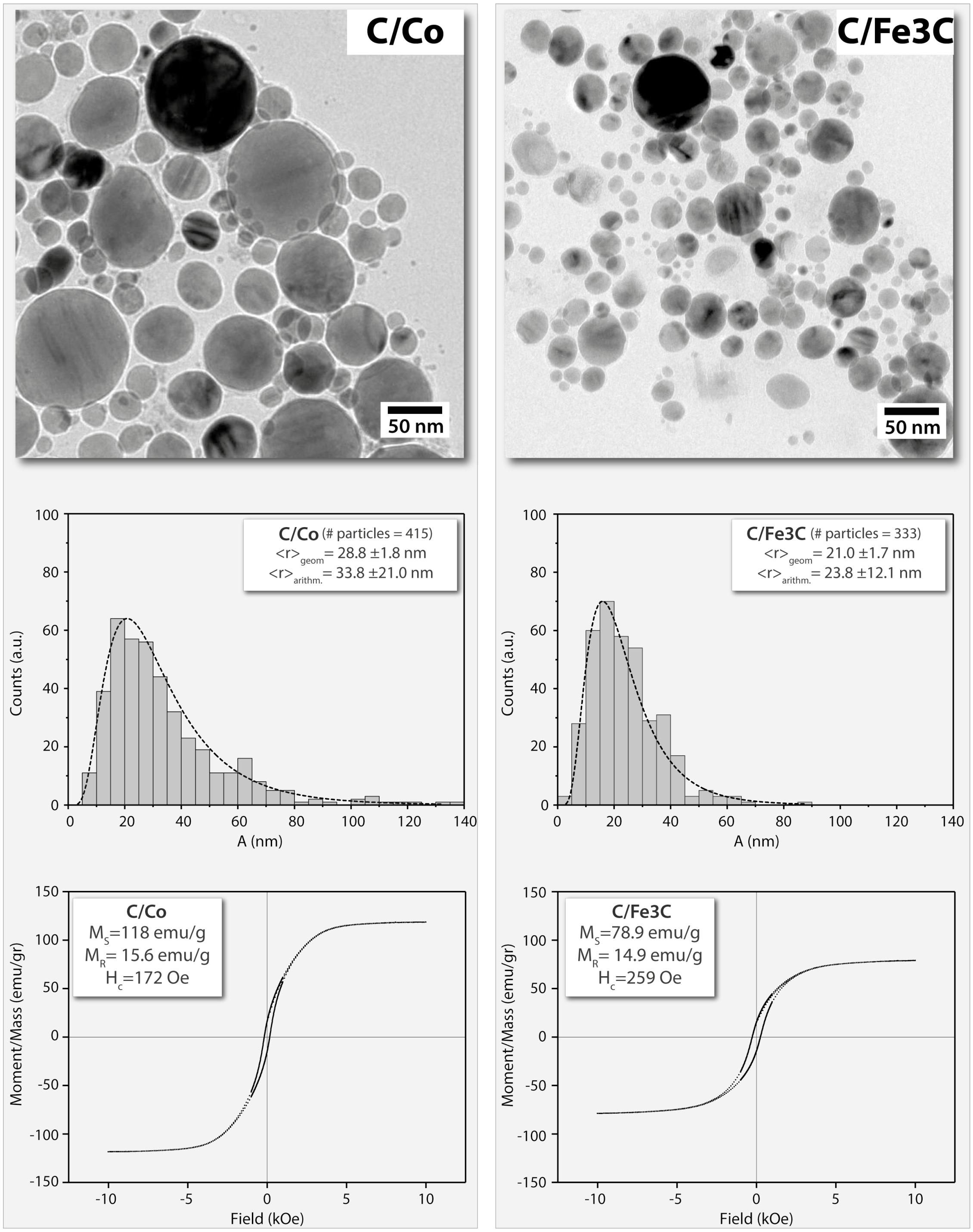

Figure S4: Scanning Transmission Electron Microscopy (STEM) images of C/Co and C/Fe3C nanoparticles. Below the STEM images for each particle type, the log-normal particle size distributions are presented along with hysteresis curves obtained by Vibrating Sample Magnetometry.

Dynamic Light Scattering Measurements

We present dynamic light scattering (DLS) measurements of C/Fe3C and C/Co particles in solutions of water and blood serum. Using DLS we investigate the hydrodynamic size distributions of the C/Fe3C and C/Co ferromagnetic particles in dispersions. For our DLS measurements we use a Zetasizer Nano (Malvern).

In particular, in Fig. S5 we present the hydrodynamic size distributions of C/Fe3C and C/Co particles obtained by DLS in water, and in Fig. S6 the DLS results for particle solutions in 10× in deionized-water-diluted bovine blood serum. We use diluted bovine serum as blood substitute since a direct measurement of particle size distributions is not possible in an optically dense medium such as non-diluted blood serum or whole blood, while diluted serum still contains significant amounts of proteins that can, e.g., lead to a protein corona on the particles.

For our measurements in both media, we apply two different sample pre-treatments: (**A**) ultrasonication for 10 s → DLS measurements; (**B**) ultrasonication for 10 s → magnetization for 120 s (neodymium magnet; 50x50x20 mm, N45; Supermagnete). The different treatments aim at identifying any effects of the pre-magnetization on particle size.

All measurements are performed using a particle concentration of 2 ppm prepared in either deionized water or 10× diluted bovine blood serum to obtain optimal DLS results. We use diluted bovine serum as blood substitute because a direct measurement of particle size distributions is not possible in an optically dense medium such as non-diluted blood serum or whole blood. Diluted serum still contains significant amounts of proteins that can, e.g., lead to a protein corona on the particles. The samples were sonicated in an ultrasonication cleaning bath for 10 s at 60 kHz. To measure DLS, 1 ml of sample was then transferred to a disposable plastic micro cuvette. DLS measurements are performed in triplicates at 25 °C.

In conclusion, we observe similar average hydrodynamic diameters for both C/Fe3C and C/Co particles in the range of 250-350 nm. In addition, we do not observe a significant effect on the hydrodynamic size following pre-magnetization in both different matrix media, i.e. water and 10x diluted bovine blood serum.

DLS in deionized H2O, concentration: 2×10^{-6} gr/ml

Run #	Treatment	Particle type	Temperature (°C)	Z-Average (d.nm)	PDI	Peak 1			Peak 2			Peak 3		
						Max d.nm	Std.dev. d.n	% Area	Max d.nm	Std.dev. d.n	% Area	Max d.nm	Std.dev. d.n	% Area
53	A	C/Fe_3C	25	405	0,006	342,3	57,4	100						
54	A	C/Fe_3C	25	450,2	0,25	351,1	61,19	100						
55	A	C/Fe_3C	25	436,9	0,353	380,7	90,42	100						
56	B	C/Fe_3C	25	319,3	0,276	351,3	111,2	93,2	91,47	16,74	6,8			
57	B	C/Fe_3C	25	329,7	0,353	370,9	123,8	91,9	79,72	13,45	6,8	5482	230,5	1,3
58	B	C/Fe_3C	25	341,8	0,358	370,2	192	97,4				5384	319,9	2,6
80	A	C/Co	25	206,2	0,156	247,6	108,4	100						
81	A	C/Co	25	226,4	0,15	266,2	105,5	100						
82	A	C/Co	25	234,1	0,184	291,8	126,2	98,2	57,59	11,46	1,8			
62	B	C/Co	25	260,1	0,226	337,7	166,5	100						
63	B	C/Co	25	286,3	0,315	389,5	250	97,2				4482	867,5	2,8
64	B	C/Co	25	309,4	0,282	386,6	210,1	95,6	41,18	9,096	1,3	4870	691,8	3,1

Z-Average: Cumulants mean

PDI: Polydispersivity index, values <0.1 very good, <0.5 ok, >0.5 refer to single peaks

Peak: Position of a maximum in intensity distribution function

% Intensity: Area under peaks compared to whole area

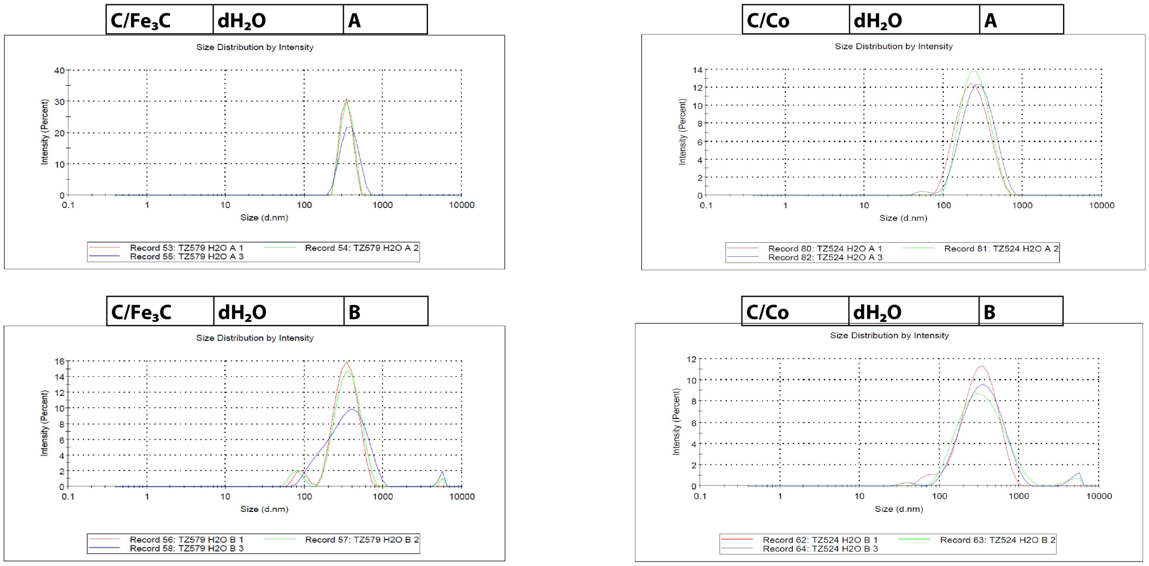

Figure S5: Hydrodynamic size distributions of C/Fe3C and C/Co particles obtained by Dynamic Light Scattering (DLS) in dionized water following different treatments: (A) with and (B) without premagnetization. All measurements are taken at a particle concentration of 2 ppm.

Magnetic field measurements

The magnetic field measurements along the magnetometer's y-axis yield higher signals since the flow tube is positioned along the custom-y-slit of the magnetometer. This is shown clearly in Fig. S7, where the y-axis signal is approximately $\times 2$ larger compared to the signal measured along the z-axis, and this is why we focus on the B_y field measurements in our work.

DLS in 10 % BOVINE SERUM, diluted in dH2O, 2×10^{-6} gr/ml

Run #	Treatment	Particle type	Temperature (°C)	Z-Average (d.nm)	PDI	Peak 1			Peak 2			Peak 3		
						Max d.nm	Std.dev. d.n	% Area	Max d.nm	Std.dev. d.n	% Area	Max d.nm	Std.dev. d.n	% Area
65	A	C/Fe_3C	25	63,85	1	279,7	91,07	68,9	30,38	7,349	17,6	7,52	1,389	12,4
66	A	C/Fe_3C	25	58,72	1	311,9	139,2	63	38,87	18,22	19,9	8,936	3,275	14,9
67	A	C/Fe_3C	25	84,24	0,721	282,4	112,3	63,7	35,64	11,26	17,7	8,724	2,299	15,6
68	B	C/Fe_3C	25	61,13	1	254,6	94,19	70,4	32,31	8,726	15,2	8,012	1,594	12,8
69	B	C/Fe_3C	25	51,93	1	339,4	154,1	68,1	38,83	12,31	18,5	8,074	1,936	13,4
70	B	C/Fe_3C	25	60,48	1	305,2	126	65,6	33,51	9,556	18,5	8,07	1,798	13,3
71	A	C/Co	25	146,5	0,564	256,8	98,24	94,2	30,36	6,791	5,8			
72	A	C/Co	25	158,4	0,55	300,2	134,5	92,9	35,7	8,568	7,1			
73	A	C/Co	25	170,3	0,602	299,6	125	87,1	32,01	6,842	5,7	8,352	1,491	5,2
74	B	C/Co	25	165,5	0,372	269,8	109,1	93,4	35,73	8,59	6,6			
75	B	C/Co	25	158,9	0,531	288,9	156,1	93,3				10,2	2,013	6,7
76	B	C/Co	25	161,6	0,584	321,6	165,1	93,7	27,76	7,052	6,3			
77	A	Serum 10 %	25	25,39	0,773	501,7	236	33,4	39,64	16,27	41,3	8,676	2,855	25,4
78	A	Serum 10 %	25	25,86	0,769	397,1	210,7	35,6	39,62	19,16	39,1	9,084	3,008	25,3
79	A	Serum 10 %	25	26,71	0,788	416,5	207,6	34,6	42,03	21,6	41,3	9,087	3,171	24,1

Z-Average: Cumulants mean
PDI: Polydispersivity index, values <0.1 very good, <0.5 ok, >0.5 refer to single peaks
Peak: Position of a maximum in intensity distribution function
% Intensity: Area under peaks compared to whole area

Very likely represent particle fraction
Very likely represent protein fraction
Exosomes or aggregates in serum, comparable low intensity to protein fraction

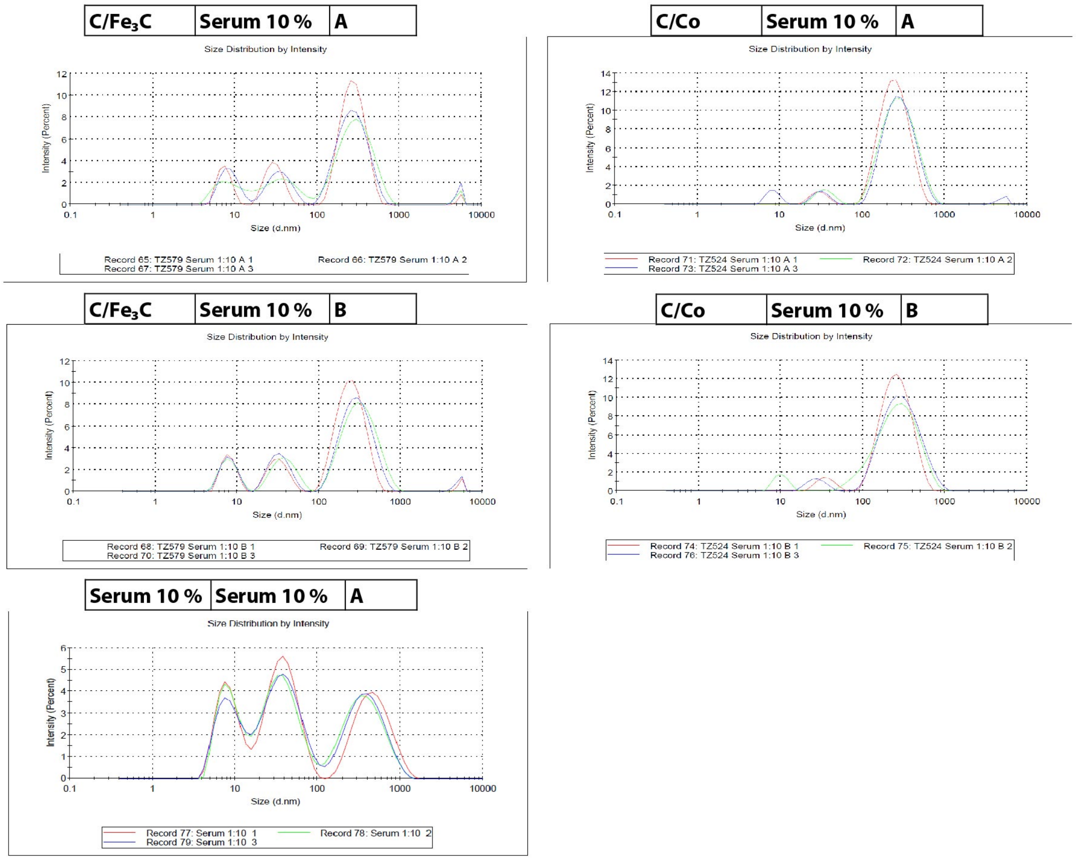

Figure S6: Hydrodynamic size distributions of C/Fe3C and C/Co particles obtained by Dynamic Light Scattering (DLS) in diluted bovine blood serum (10× in deionized water) following different treatments: (A) with and (B) without premagnetization. All measurements are taken at a particle concentration of 2 ppm prepared in 10× diluted bovine blood serum to obtain optimal DLS results.

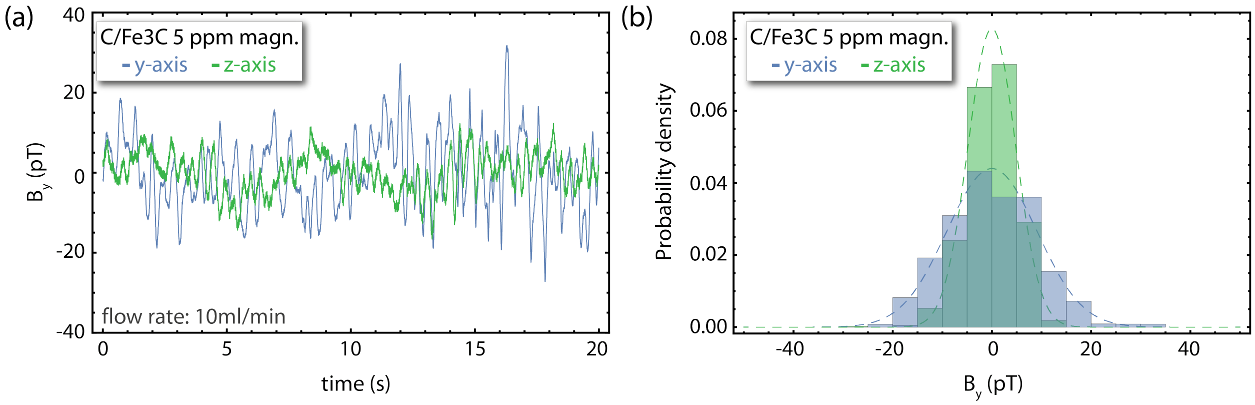

Figure S7: (a) Real-time magnetic field measurements along the y- and z-axis of the magnetometer for a 5 ppm C/Fe3C:water solution. (b) Histogram analysis of the measurements shown in (a). The square-root of the magnetic-field-variance is 9.0 pT and 4.8 pT for the y- and z-axis, respectively.

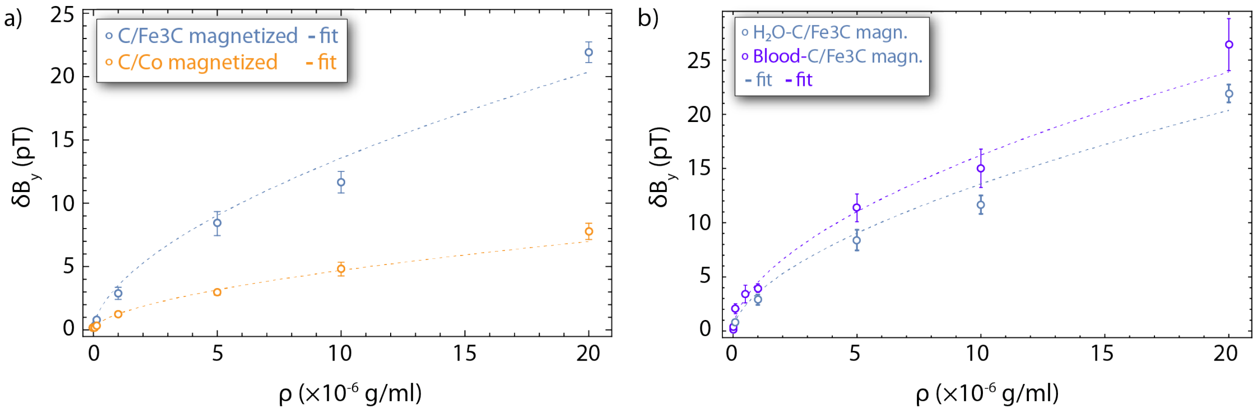

Figure S8: Magnetic field variance signal for different concentrations of the C/Fe3C, C/Co, under pre-magnetized conditions, in water solutions (the same data are presented in Fig. 3 of the main text in a logarithmic scale). All measurements are realized under constant flow conditions (10 ml/min). (a) A power-law fit (ρ^n) yields the dependence between the observed magnetic field fluctuations and the particle concentrations to be $n = 0.59$ for the C/Fe3C measurements in water and $n = 0.57$ for the C/Co measurements in water. (b) A power-law fit yields the dependence between the observed magnetic field fluctuations and the particle concentrations to be $n = 0.56$ for the C/Fe3C measurements in blood.

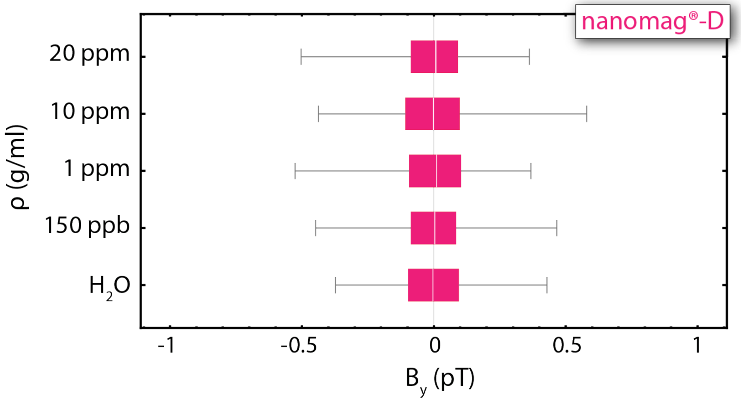

Figure S9: A box-and-whisker plot of the distribution of the acquired values for each measurement performed using Micromod's nanomag®-D, plain, 130 nm diameter, superparamagnetic nanoparticles. The white line represents the median marker, the purple boxes represent the upper and lower 25% quantiles, while the gray bars represent the maximum and minimum acquired data points (these bars include data far outliers). The baseline measurement realized with pure water flowing in front of the detector is also shown for comparison. These data are also presented in Fig. 3 of the main text.

Magnetic field estimations

The maximum strength of the magnetic field produced by a ferromagnetic particle at a distance r is proportional to its net magnetic dipole moment $\mathbf{m}$ and it scales as a function of distance as (in Gaussian units) (see Maser et al. [2011]):

$$B_{\text{particle}} \propto \frac{|\mathbf{m}|}{r^3}. \tag{1}$$

Using the characterisation data for the particles used in this work (Fig. S4), and Eq. 1, we can estimate the detection limit in terms of particle concentration in our system.

The C/Fe3C ferromagnetic particles have a density of approximately 7.9 g/ml, a mean diameter of ∼24 nm, and a magnetization saturation of ∼80 emu/g. Therefore, the net magnetic moment of a C/Fe_3C nanoparticle can be estimated to be:

$$|\mathbf{m}| \simeq 80 \times \frac{4}{3}\pi\,(12 \times 10^{-7})^3 \times 7.9 \simeq 4.6 \times 10^{-15}\,\text{emu}. \tag{2}$$

The magnetic field sensor (i.e. the vapor cell) is located at a distance of approximately ∼6 mm from the centre of the tubing. Thus, the maximum measured magnetic field produced by a single C/Fe_3C ferromagnetic particle at the sensor is:

$$B_{\text{particle}} \simeq \frac{4.6 \times 10^{-15}}{(6 \times 10^{-1})^3} \times 4\pi \times 10^{-4}\,\text{T} \;\simeq\; 2.7 \times 10^{-17}\,\text{T}. \tag{3}$$

In Fig. 3 of the main text we present the relationship between the detected magnetic-field variance and the particle concentration. In particular, we observe an approximate square-root-dependence between the measured magnetic field variance and the particle concentration (see also Fig. S8 in the supplementary materials). Therefore, we can estimate the detection limit in our measurements by using the following relationship: $\delta\text{B} \propto \sqrt{N} \cdot B_{\text{particle}}$, where N is the number of particles in the sensing volume. The integrated sensitivity for our measurements of magnetic field variance (for magnetic fields along the y-axis) in water and blood solutions is approximately 200 fT. Thus, our sensitivity limit corresponds to approximately $\sim 6 \times 10^7$ particles. Using Eq. 3 we can also estimate the sensing volume limit, assuming a minimum detectable variance signal of ∼200 fT and $N = 6 \times 10^7$ particles. The approximate sensing volume limit is found to be ∼0.2 ml.

From our measurements (Fig. 3, main text) we estimate the sensitivity of our sensing protocol at the 5 ppb level (corresponding to 5×10^{-9} g/ml) for the C/Fe3C nanoparticles in water solutions. For sensing volumes of ∼0.2 ml, this sensitivity limit corresponds to $\sim 2 \times 10^7$ particles. Thus, the observed signals and results obtained during our measurements are consistent with the estimates produced using Eq. 3.

Using similar calculations, we can estimate expected signals for particles of different sizes. For example, a single ferromagnetic particle of ∼60 nm in diameter, with a density of 7.9 g/ml, and a saturation magnetization of 80 emu/g, will produce a maximum magnetic field at the sensor of ∼ 0.4 fT. Thus, if our signals were originating from particles of ∼ 60 nm in diameter, the 200 fT integrated sensitivity of our measurements would correspond to $\sim 2.3 \times 10^5$ particles, which corresponds to 10 ppt particle-concentration sensitivities. Similarly, a ferromagnetic particle of ∼300 nm in diameter, can produce a maximum magnetic field at the sensor of ∼ 52fT.

The above simplified calculation are instructive in showing that using a high performance optical magnetometer and our measurement technique, the detection of particles as small 24 nm at the 5 ppb levels is possible. It also becomes apparent from our calculation, that our measurement technique is sensitive enough to detect single ferromagnetic particles with sizes equal or larger than 300 nm (a result supported by the results shown in Refs. 30, 31, 46, 47).

Finally, we note here one possible deviation from the simplified calculation presented here. To magnetically saturate the ferromagentic particles would require a pre-magnetization step at fields as high as 4 T. The magnetic system used in our experiments to pre-magnetize the particles is a a 2 T Halbach array magnet, and thus we are most likely not achieving maximum saturation magnetization for the full sample.

Flow Rates

In Figure S10 we present magnetic field measurements under continuous flow of water solutions of 10 ppm C/Fe3C nanoparticles for 5 different flow rates through setup (A) (see main text, Fig. 1). For flow rates equal or higher than 10 ml/min, we do not observe a strong flow-dependence on the observed signal. For lower flow rates, as is apparent in the data acquired for a flow rate of 5 ml/min, the measured signal becomes smaller. We attribute this effect to the ring magnet incorporated in the measurement apparatus (see discussion and schematic in main article). For slow flow rates (<10 ml/min) the ring magnet results in magnetically separating nanoparticles from the flow, and thus, it reduces the concentration of particles reaching the magnetometer, yielding smaller signals. Note the expected time dependence on the flow rate of the observed magnetic field fluctuations (i.e. slower fluctuations for slower flow rates).

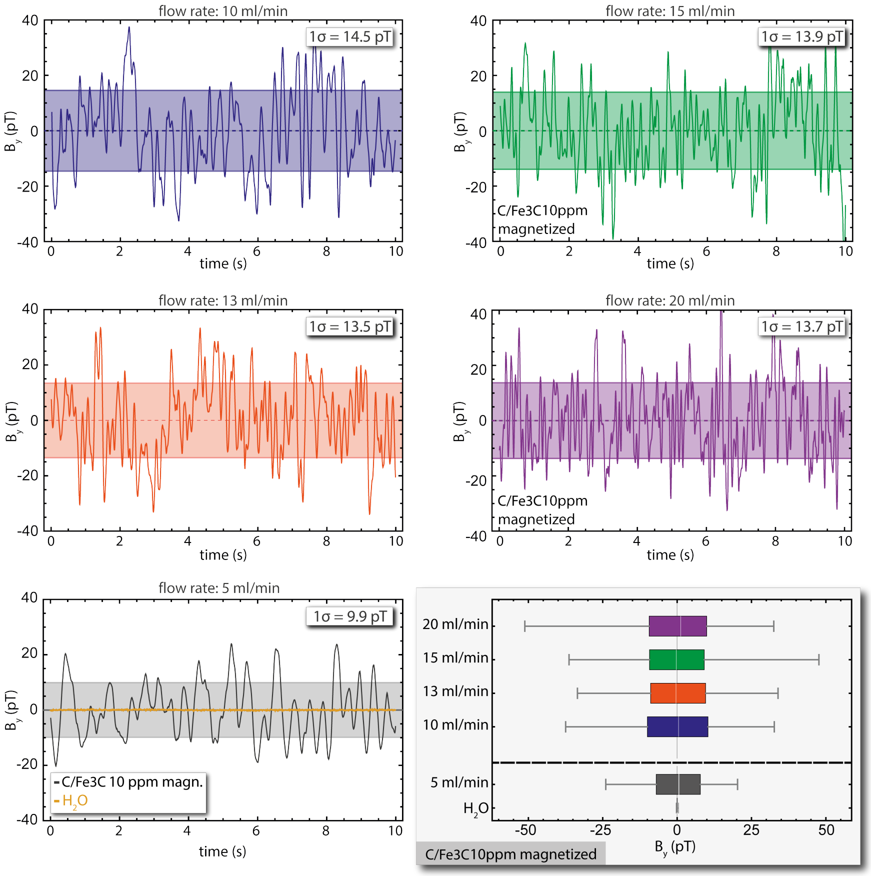

Figure S10: Magnetic field measurements along the y-axis, for water solutions of 10 ppm C/Fe3C nanoparticles, under constant flow conditions, for different flow rates. The shaded areas (colored boxes) in each graph are within one standard deviation of the mean (68% of the magnetic field fluctuation distributions). Box-and-whisker plots of the distributions of the acquired values for each measurement are also shown. For each measurement, the white line represents the median marker, the colored boxes represent the upper and lower 25% quantiles, while the gray bars represent the maximum and minimum acquired data points (these bars include data far outliers). For comparison, we present also the box-and-whisker plot for the measurement-baseline of the magnetometer when only pure water is run through the system (the real-time trace data for pure water are presented together with the measurement for the 5 ml/min flow rate).

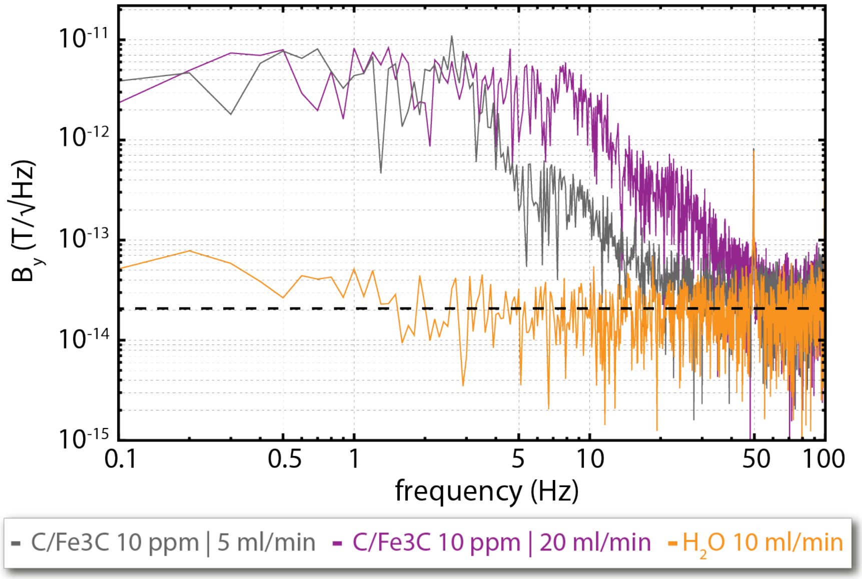

Figure S11: Magnetic field power spectra for two different flow rates (Fig. S10) for water solutions of 10 ppm C/Fe3C nanoparticles.

Magnetic separation

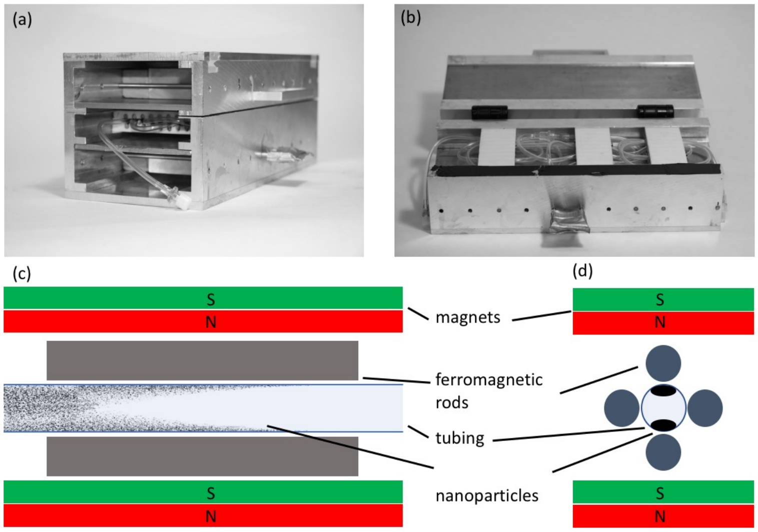

Figure S12: (a) and (b) Photographs of the high gradient magnetic separator. (c) Schematic of high gradient magnetic separator [see Ref. Herrman et al. [2011]]. Ferromagnetic rods are employed to increase the field gradient Bockenfeld et al. [2010]. Particles are separated from flow and stick to the tube walls. The tubing is passing several separation-regions within the separator.

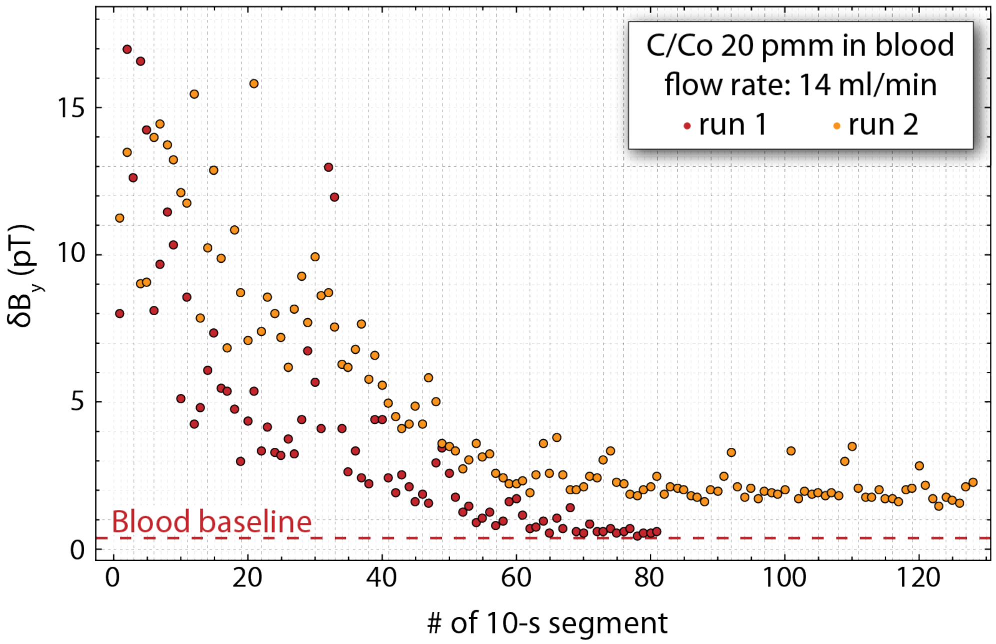

Figure S13: Two different runs for nanoparticle magnetic separation in porcine blood. In both experimental runs, magnetic separation is successful and observable via our analysis, although for the second run a higher noise floor was observed which we attribute to low-frequency drifts related to vibration noises.

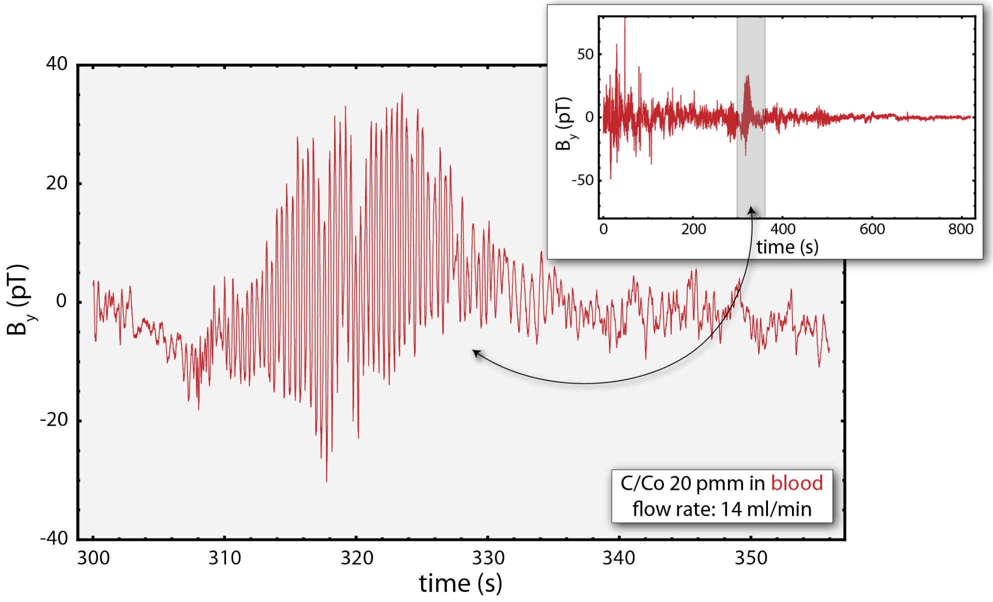

Figure S14: Particle aggregation as observed in the blood separation data presented in Fig. 4 of the main article. Under typical experimental conditions (for a given particle solution and constant flow rate) we observe random magnetic field oscillations (see Fig. 2 of the main text, Fig. S8, and signals shown here for the time window ∼300-310 s). In the time window ∼310-330 s we observe a fast oscillatory signal, of large, and relatively constant, amplitude. We attribute this signal to particle aggregates, which may form a chain of ferromagnetic particles.

References

D. Bockenfeld, H. Chen, M. D. Kaminski, A. J. Rosengart, and D. Rempfer. A parametric study of a portable magnetic separator for separation of nanospheres from circulatory system. *Separation Science and Technology*, 45(3):355–363, 2010.

I. K. Herrman, R. E. Bernabei, M. Urner, R. N. Grass, B. Beck-Schimmer, and W. J. Stark. Device for continuous extracorporeal blood purification using target-specific metal nanomagnets. *Nephrology Dialysis Transplantation*, 26(9):2948, 2011.

D. Maser, S. Pandey, H. Ring, M. P. Ledbetter, S. Knappe, J. Kitching, and D. Budker. Note: Detection of a single cobalt microparticle with a microfabricated atomic magnetometer. *Review of Scientific Instruments*, 82(8):086112, 2011.
